# Cryopreserved clumps of mesenchymal stem cell/extracellular matrix complexes retain osteogenic capacity and induce bone regeneration

**DOI:** 10.1186/s13287-018-0826-0

**Published:** 2018-03-21

**Authors:** Souta Motoike, Mikihito Kajiya, Nao Komatsu, Manabu Takewaki, Susumu Horikoshi, Shinji Matsuda, Kazuhisa Ouhara, Tomoyuki Iwata, Katsuhiro Takeda, Tsuyoshi Fujita, Hidemi Kurihara

**Affiliations:** Department of Periodontal Medicine, Applied Life Sciences, Institute of Biomedical & Health Sciences, Graduate School of Biomedical & Health Sciences, Hiroshima University, 1-2-3, Kasumi, Minami-ku, Hiroshima, Japan

**Keywords:** Artificial scaffold free, Bone regeneration, Cryopreservation, C-MSC, MSCs

## Abstract

**Background:**

Three-dimensional (3D) cultured clumps of mesenchymal stem cell (MSC)/extracellular matrix (ECM) complexes (C-MSCs) consist of cells and self-produced ECM. C-MSCs can regulate cellular functions in vitro and can be grafted into a defect site without an artificial scaffold to induce bone regeneration. Long-term cryopreservation of C-MSCs, which can enable them to serve as a ready-to-use cell preparation, may be helpful in developing beneficial cell therapy for bone regeneration. Therefore, the aim of this study was to investigate the effect of cryopreservation on C-MSCs.

**Methods:**

MSCs isolated from rat femurs were cultured in growth medium supplemented with ascorbic acid. To obtain C-MSCs, confluent cells that had formed on the cellular sheet were scratched using a micropipette tip and were then torn off. The sheet was rolled to make a round clumps of cells. The C-MSCs were cryopreserved in cryomedium including 10% dimethyl sulfoxide.

**Results:**

Cryopreserved C-MSCs retained their 3D structure and did not exhibit a decrease in cell viability. In addition, stem cell marker expression levels and the osteogenic differentiation properties of C-MSCs were not reduced by cryopreservation. However, C-MSCs pretreated with collagenase before cryopreservation showed a lower level of type I collagen and could not retain their 3D structure, and their rates of cell death increased during cryopreservation. Both C-MSC and cryopreserved C-MSC transplantation into rat calvarial defects induced successful bone regeneration.

**Conclusion:**

These data indicate that cryopreservation does not reduce the biological properties of C-MSCs because of its abundant type I collagen. More specifically, cryopreserved C-MSCs could be applicable for novel bone regenerative therapies.

## Background

Mesenchymal stem cells (MSCs) are self-renewing multipotent progenitor cells that have attracted considerable scientific and medical attention for many years as an effective tissue regenerative cell therapy [1–4]. In particular, bone marrow-derived MSCs are highly investigated stem cells for bone regeneration in basic and clinical studies [5]. It is widely accepted that the implantation of bone marrow-derived MSCs promotes bone regeneration at the sites of defects. However, there still remain obstacles to be overcome in applying these cells for established bone regenerative medicine. At present, MSCs isolated from patient bone marrow are expanded ex vivo and then mixed with biocompatible artificial scaffold to graft the cells into the defect site. This process requires a prolonged culture period which results in increased contamination risks and culture costs. In addition, despite recent advances, clinical application of artificial scaffolds still harbors several limitations, including biodegradability and unfavorable host inflammation and immunological reaction [6, 7].

To address these problems, we have recently generated three-dimensional (3D) clumps of MSC/extracellular matrix (ECM) complexes (C-MSCs), which consist of cells and self-produced ECM [8]. C-MSCs can be grafted into bony lesions without artificial scaffolds to induce successful bone regeneration [8, 9], suggesting the avoidance of the problems regarding the usage of artificial scaffolds described above. Moreover, we have also reported that xenografts of human C-MSCs treated with interferon (IFN)-γ induced bone regeneration in a mouse calvarial defect model because of its highly regulated immunomodulatory capacity [10]. This fact indicated the availability of allogenic C-MSCs for clinical bone regenerative cell therapy, which can eliminate the autologous MSC isolation and expansion process. However, even though C-MSCs seem to be promising for clinical bone regenerative cell therapy, their preparation process is inevitably time consuming.

Cryopreservation, which maintains the cell viability and function of bioengineered cellular constructs, is a significant research avenue for successful tissue engineering in regenerative medicine [11]. The development of cryo-banked materials will enable us to supply the cellular product at the time when the patient needs it. Moreover, the materials can provide adequate quality control and standardization of the same cell preparation at different times when the cellular product is needed. Briefly, if cryopreserved C-MSCs retain their 3D structure, cell viability, and osteogenic properties, C-MSCs will take an important step toward their clinical application for bone regenerative medicine because the C-MSC preparation process can be omitted immediately before its transplantation and we will have standardized material on demand.

This novel cell therapy using cryopreserved C-MSCs could be implemented through optimized easy cryopreservation procedures. In general, cryopreservation of cells or tissues is carried out by two techniques, either vitrification or slow freezing in the presence of a cryoprotectant, such as dimethyl sulfoxide (DMSO). Although vitrification is well known to show beneficial cytoprotective effects in various types of cells, its inherent problems include the difficulty of large-scale processing and risk of contamination from liquid nitrogen [12, 13]. On the other hand, slow freezing is a well-established traditional approach for cryopreservation of MSCs as cell suspensions [14–16]. Importantly, this procedure can handle a large number of samples easily, which makes it more clinically relevant. Moreover, a recent study revealed that this slow freezing is an effective technique for not only the cells but also for ECM preservation [17].

Accordingly, this present study investigated whether cryopreservation could affect the structural integrity, cell viability, and osteogenic property of C-MSCs. In addition, we also tested the stability of other 3D cell culture systems, including cell sheets and cell spheroids, in cryopreservation and explored the underlying mechanism that plays a protective role against damage by cryopreservation in these cellular constructs. Moreover, the bone regenerative capacity of cryopreserved C-MSCs was tested in a rat calvarial defect model.

## Methods

### Rat C-MSC preparation and culture

**Fig. 1:**
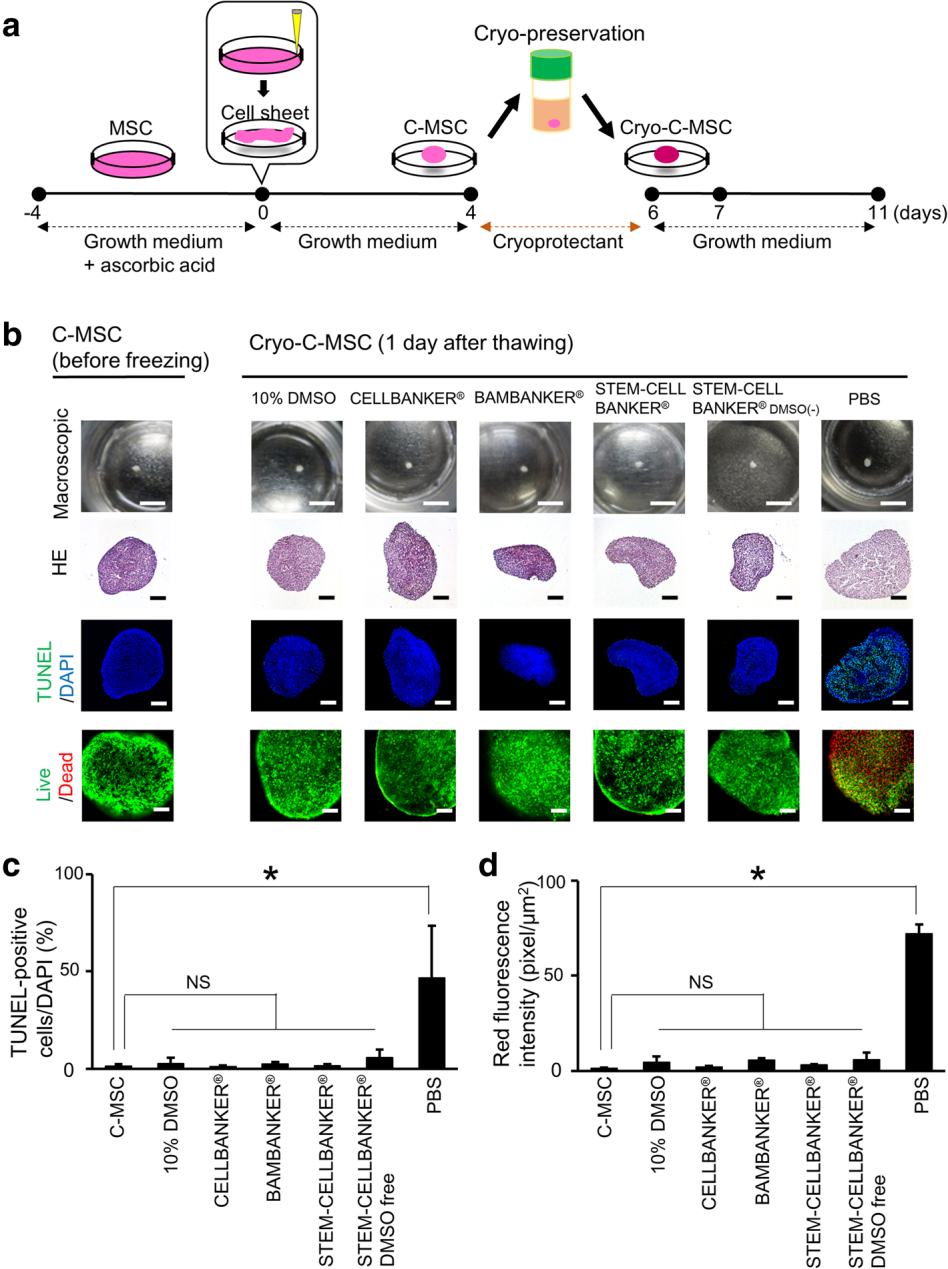
Cryopreservation does not reduce cell viability in C-MSCs. **a** Schematic figure of the preparation and cryopreservation of clumps of mesenchymal stem cell/extracellular matrix complexes (C-MSCs). **b** C-MSCs or cryopreserved C-MSCs (Cryo-C-MSC) with each cryopreservation solution or phosphate-buffered saline (PBS) were prepared as described in the Methods. Upper panels show macroscopic images of C-MSCs or cryopreserved C-MSCs in 24-well culture plates. Scale bars = 4 mm. Serial sections were stained with hematoxylin and eosin (H&E) and TUNEL as indicated. In addition, to monitor the cell viability, live (green) and dead cells (red) were distinguished using a LIVE/DEAD Viability/Cytotoxicity kit. Scale bars = 200 μm. **c** The percentage of TUNEL-positive apoptotic cells. Values represent means ± SD of four cultured C-MSCs. **d** The red fluorescence intensity of EthD-1, indicating dead cells. Values represent means ± SD of four cultured C-MSCs. Similar results were obtained from three independent experiments. *p < 0.05, by ANOVA with Tukey’s test. DAPI 4′,6-diamidino-2-phenylindole, DMSO dimethyl sulfoxide, NS not significant

Male F344/DuCrlCrlj rats (Charles River Laboratories Japan, Yokohama, Japan) were employed in this study after approval had been obtained from the Animal Care Committee of Hiroshima University. Rat MSCs at the third passage were obtained from the bone marrow of femurs taken from 3-week-old rats, as described previously [8]. The cells were maintained in Dulbecco’s modified Eagle’s medium (DMEM; Sigma, Steinheim, Germany) supplemented with 10% fetal bovine serum (FBS; Sigma), 100 U/mL penicillin (Sigma), and 100 μg/mL streptomycin (Sigma), and then C-MSCs were prepared as reported previously with minor modification [8]. Briefly, MSCs were seeded at a density of 2.0 × 10^5^ cells/well in 24-well plates (Corning, Corning, NY) and cultured with high-glucose DMEM (Sigma) supplemented with 10% FBS, 100 U/mL penicillin, 100 μg/mL streptomycin (growth medium), and 50 μg/ml l-ascorbic acid (Sigma) for 4 days. To obtain C-MSCs, confluent cells that had formed on the cellular sheet, consisting of self-produced ECM, were scratched using a micropipette tip and then torn off. The MSC/ECM complexes detached from the bottom of the plate in a sheet shape were transferred to a 24-well ultra-low-binding plate (Corning) and rolled up to make a round clumps of cells. After a 1-day incubation, 0.8–1.2 mm diameter C-MSCs were obtained and maintained in growth medium for 4 days (Fig. 1a).

### Preparation of rat MSC spheroids

MSC spheroids were generated as reported previously with minor modifications [18]. Briefly, the cells were seeded at a density of 2.0 × 10^5^ cells/well in ultra-low-binding 24-well plates (Iwaki, Chiba, Japan) and cultured with growth medium in the presence or absence of 50 μg/mL l-ascorbic acid for 4 days. Then, 0.6–0.8 mm diameter MSC spheroids were obtained.

### Cryopreservation study

Standard cryomedium (DMEM + 20% FBS + 10% DMSO), four commercial cryopreservation solutions (CELLBANKER®, Juji Field, Tokyo, Japan; BAMBANKER®, Jappan Genetics, Tokyo, Japan; STEM-CELLBANKER®, Takara, Tokyo, Japan; or STEM-CELLBANKER® DMSO free, Takara), or phosphate-buffered saline (PBS) were employed in this study. One C-MSC or MSC spheroid precultured for 4 days or a cellular sheet obtained after micropipette scratching, as described above, was soaked in 500 μL cryoprotectant solution and then transferred to a cryotube vial (Nunc cryotube®, Thermo Scientific, Waltham, MA). The samples were then placed directly into a deep-freezer set at −80 °C. After 2 days of cryopreservation, some samples were placed in a 37 °C water bath for rapid thawing until almost no ice was detectable. The C-MSCs, MSC spheroids, and cellular sheets were transferred into a 24-well culture plate containing growth medium and washed thoroughly to remove cryomedium from the samples. C-MSCs without cryopreservation were set as a control. For the long-term cryopreservation study, the samples were transferred from a deep-freezer to a liquid nitrogen tank and stored for 6 months.

### Cell viability assay

To measure the cellular recovery from cryopreservation, the cell viability of C-MSCs was assessed using a LIVE/DEAD Viability/Cytotoxicity kit (Invitrogen, Carlsbad, CA). Briefly, the C-MSCs were washed with PBS and stained by incubation in PBS containing 2 μM calcein-AM and 1 μM ethidium homodimer (EthD-1) for 40 min at 37 °C. The samples were then placed onto a cover glass and visualized using a Zeiss LSM 510 laser scanning confocal microscope (Zeiss Microimaging, Inc., Thornwood, NY). Living cells stained with calcein-AM exhibited a green color, whereas dead cells stained with EthD-1 fluoresced red when examined using a fluorescence microscope. Pixel analysis was performed using the Java-based image processing software ImageJ (NIH, Bethesda, MD).

### Histological and immunofluorescence analysis

Cultured samples with or without cryopreservation were fixed with 1% paraformaldehyde and embedded in paraffin. Five-micrometer serial sections were prepared. The specimens were then stained with hematoxylin and eosin (H&E) and observed using a light microscope. For type I collagen staining, the samples were treated with 1% bovine serum albumin (BSA) and 0.1% Triton-X100 in PBS to block nonspecific staining. These sections were then treated with a rabbit anti-rat type I collagen IgG antibody (1:500; Abcam, Cambridge, MA) at 4 °C overnight. After washing three times with PBS for 5 min, samples were incubated for 1 h with an Alexa Fluor 488® goat anti-rabbit IgG antibody (1:200; Invitrogen) at room temperature. Nuclei and F-actin were counterstained with 4′,6-diamidino-2-phenylindole (DAPI; Invitrogen; 5 μg/mL) and Alexa Fluor 594® phalloidin (1:50; Invitrogen), respectively. To detect apoptotic cells, the sectioned samples were assessed using a DeadEnd™ Fluorometric TUNEL System (Promega, Madison, WI) [19] in accordance with the manufacturer’s instructions. Fluorescence signals were detected using a Zeiss LSM 5 PASCAL laser scanning confocal microscope.

### Flow cytometric analysis for the cell surface antigens

C-MSCs or cryopreserved C-MSCs were dissociated using a gentleMACS Dissociator (Milteny Biotech, Bergish-Gladbach, Germany). The dissected samples were filtered through sterile 70-μm nylon cell strainers (BD, Franklin Lakes, NJ) to obtain cell suspensions. The cells were then incubated with a mouse monoclonal anti-CD90 IgG antibody (BD; #2E11), mouse monoclonal anti-CD73 IgG antibody (BD; #5F/B9), mouse monoclonal anti-CD34 IgG antibody (Santa Cruz, Dallas, TX; #ICO115), and mouse monoclonal anti-CD45 IgG antibody (BD; #OX-1) for 1 h at room temperature. The cells were then incubated with a fluorescein isothiocyanate (FITC)-conjugated horse anti-mouse-IgG antibody (Vector Laboratory, Burlingame, CA) for 30 min at room temperature. The expression profile of each molecule was determined with a FACScan flow cytometer (BD) using Cell Quest software (BD).

### Osteogenic induction

C-MSCs or C-MSCs cryopreserved in standard cryomedium were cultured with growth medium or osteoinductive medium (OIM; growth medium supplemented with 10 nM dexamethasone (Sigma), 50 μg/mL l-ascorbic acid (Sigma), and 10 mM β-glycerophosphate (Sigma)) for 5 days. The osteogenic gene expression level and calcium content were then measured using alkaline phosphatase (ALP) activity and a calcium content assay kit, respectively. Briefly, C-MSCs were lysed with 0.025% Triton X solution. The cell lysate was centrifuged at 15,000 rpm for 15 min and ALP activity was measured in the supernatant with a Lab Assay ALP kit (Wako, Osaka, Japan) in accordance with the manufacturer’s instructions. The absorbance of the resultant solution was measured at 405 nm using a microplate reader. For the measurement of calcium content, cultured C-MSCs were lysed using 5 N hydrochloric acid solution with sonication. Dissolved calcium was then measured using a Calcium E-test kit (Wako). The absorbance of the resultant solution was measured at 610 nm using a microplate reader.

### Surgical procedure

To assess the bone regenerative properties of C-MSCs, a rat calvarial defect model was employed [8]. Eight-week-old F344 male rats were anesthetized with an intraperitoneal injection of 20% ethyl carbamate (30 mg/kg body weight). The skin at the surgical site was shaved and disinfected, and a sagittal skin incision was made from the occipital to the frontal bone. The skin flap, including the periosteum, was then dissected and elevated. Avoiding the cranial suture, calvarial defects of 1.6 mm diameter was created in the parietal bone. Cryopreserved C-MSCs kept for 2 days in deep-freezer or for 6 months in liquid nitrogen were cultured with growth medium for 5 days and were transplanted into the defect directly without any artificial scaffold. In addition, the implantation of C-MSCs maintained in growth medium for 5 days without cryopreservation was set as a control. The skin incision was then closed using 4–0 silk sutures.

### Microcomputed tomography analysis

The animals were sacrificed 28 days after surgery and the cranial region was imaged using SkyScan1176 in vivo microcomputed tomography (μCT; Bruker, Billerica, MA). Three-dimensional reconstructions were generated using CTVOL software (Bruker). The volume of newly formed bone inside the bone defect was determined using CT-An software (Bruker).

### Tissue preparation and histological analysis

Rats were sacrificed 28 days after surgery. Calvarial bones were collected, fixed with 4% paraformaldehyde overnight, and decalcified with 10% ethylenediaminetetraacetic acid (EDTA; pH 7.4) for 14 days. After decalcification, the specimens were dehydrated through graded ethanol, cleared with xylene, and embedded in paraffin. Serial sections (5 μm) were cut in the frontal plane. These sections, representing the central portion of the bone defect, were stained with H&E and observed using a light microscope.

### Statistical analysis

Data were analyzed by analysis of variance (ANOVA) or Student’s *t* test. Values of *p* < 0.05 or *p* < 0.01 were considered significant.

## Results

### Cryopreserved C-MSCs show aggregable cell viability

C-MSCs consisted of cells and ECM, and TUNEL staining showed few apoptotic cells in C-MSCs, as reported previously (Fig. 1b, left panel) [8]. In addition, calcein AM (green) and EthD-1 (red) staining indicated that the majority of the cells in C-MSCs were viable and few dead cells were present (Fig. 1b, left panel). It is noteworthy that all C-MSCs cryopreserved with standard cryomedium (DMEM + 20%FBS + 10% DMSO) or four commercial cryopreservation solutions (CELLBANKER®, BAMBANKER®, STEM-CELLBANKER®, or STEM-CELLBANKER® DMSO free) retained a round shape composed of ECM, and the number of dead cells did not increase (Fig. 1b–d), whereas cryopreservation using PBS caused high levels of cell death in C-MSCs (Fig. 1b–d). These findings suggested that cryopreservation of C-MSCs using any type of cyroprotectant did not decrease cell viability. Since all tested cryopreservation solutions showed similar and desirable cytoprotective effects, in the following study we focused on the standard cryomedium of which composition was clearest.

### Cell surface marker expressions and osteogenic properties in cryopreserved C-MSCs

**Fig. 2:**
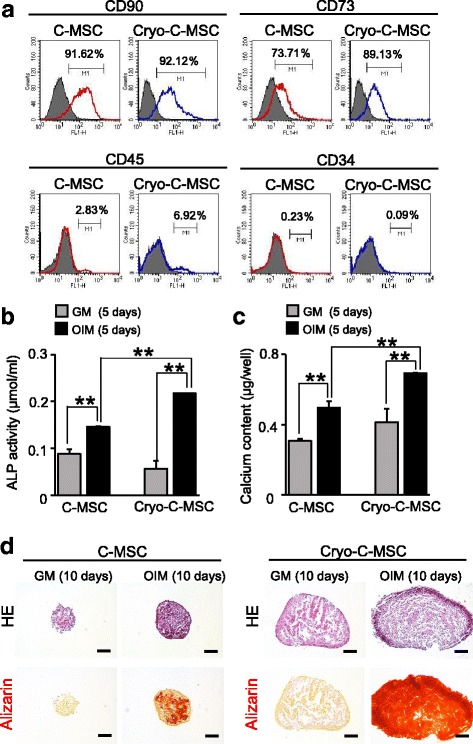
Cell surface marker expression levels and osteogenic properties of cryopreserved C-MSCs. **a** Cell surface marker expression levels in clumps of mesenchymal stem cell/extracellular matrix complexes (C-MSCs) or cryopreserved C-MSCs (Cryo-C-MSC) were monitored by flow cytometry as described in the Methods. The open histogram with red or blue lines indicates CD90-, CD73-, CD45-, and CD34-positive cells. The IgG control is shown as a solid histogram. **b**–**d** C-MSCs or Cryo-C-MSCs were cultured in growth medium (GM) or osteoinductive medium (OIM) for the indicated culture period. Alkaline phosphatase (ALP) activity (**b**) and calcium content (**c**) in cultured C-MSCs were monitored as described in the Methods. Data are the mean ± SD of four cultures. **p < 0.01, by *t* test. Similar results were obtained from three experiments. **d** Serial sections were stained with hematoxylin and eosin (HE; upper panels) and Alizarin Red (lower panels) as indicated. Scale bars = 200 μm. The photographs are representative of three independent experiments

Next, we investigated the effect of cryopreservation on stem cell phenotypic markers and the osteogenic differentiative potential of C-MSCs. Both C-MSCs and cryopreserved C-MSCs expressed similar levels of standard rat MSC markers (CD73 and CD90) but lacked the negative markers CD45 and CD34 (Fig. 2a). ALP activity and calcium content were significantly increased in C-MSCs cultured in OIM for 5 days (Fig. 2b, c). Consistent with the calcium content, OIM treatment markedly elevated calcium deposition identified by Alizarin Red staining in C-MSCs at day 10 (Fig. 2d). Importantly, cryopreserved C-MSCs cultured with OIM also demonstrated higher levels of ALP activity and calcium content (Fig. 2b, c) and a remarkable staining pattern of Alizarin Red (Fig. 2d). In addition, cryopreserved C-MSCs cultured with OIM demonstrated higher levels of ALP activity, calcium content, and mineral deposition than those of C-MSCs (Fig. 2b–d). These findings indicated that cryopreserved C-MSCs possessed higher osteogenic differentiation properties compared with C-MSCs.

### Cryopreservation causes severe damage to cell sheets and cell spheroids

**Fig. 3:**
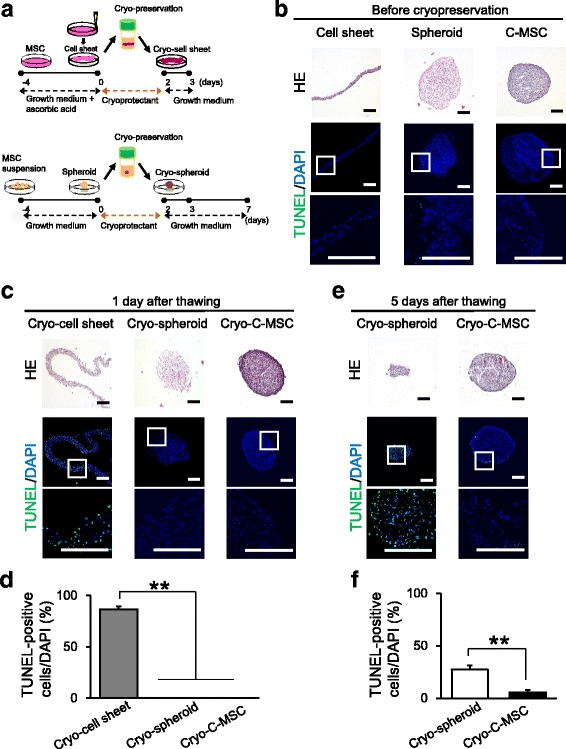
Cryopreservation damage to other 3D mesenchymal stem cell (MSC) compositions, including cell sheets and spheroids. Cell sheets, cell spheroids, or clumps of mesenchymal stem cell/extracellular matrix complexes (C-MSCs) were generated and cryopreserved as described in the Methods. **a** Schematic figure of cell sheets and spheroids preparation and cryopreservation. (For C-MSC cryopreservation, see Fig. 1a.) Histological analysis of each cell construct before cryopreservation (**b**) or at 1 (**c**) and 5 days (**e**) after thawing. Serial sections were stained with hematoxylin and eosin (HE) and TUNEL as indicated. Scale bars = 200 μm. The percentage of TUNEL-positive apoptotic cells at 1 day (**d**) or 5 days (**f**) after thawing. Values represent means ± SD of four cultured cell constructs. Similar results were obtained from three independent experiments. **p < 0.01, by ANOVA with Tukey’s test. DAPI 4′,6-diamidino-2-phenylindole

Since artificial scaffold-free 3D cell culture systems, cell sheets, and cell spheroids are well recognized [20, 21], we next examined whether these scaffold-free cell culture systems can also be cryopreserved like C-MSCs. Compared with normal cell sheets, cryopreserved sheets had a frayed shape and almost all cells were apoptotic, as indicated by TUNEL staining (Fig. 3b–d). Cell spheroids showed a round shape similar to C-MSCs before cryopreservation (Fig. 3b). However, cryopreserved cell spheroids collapsed 1 day after thawing (Fig. 3c), and dissociation was obvious at day 5 (Fig. 3e). Along with the collapse of the spheroidal structure, the number of TUNEL-positive apoptotic cells increased in cryopreserved cell spheroids by 5 days after thawing (Fig. 3e, f). In contrast, cryopreserved C-MSCs retained their shape until 5 days after thawing, and only a few apoptotic cells were detectable at the periphery of the cell clumps (Fig. 3e). These findings suggested that cryopreservation damaged the structure of the cellular sheet and spheroid which, in turn, resulted in the induction of cell death in those cellular constructs. On the other hand, C-MSCs that possess condensed ECM structure by folding the thin cell sheet retained the structure of the cellular construct and did not show extensive cell death after cryopreservation.

### Abundant type I collagen protects the structure of 3D cellular constructs from damage by cryopreservation

**Fig. 4:**
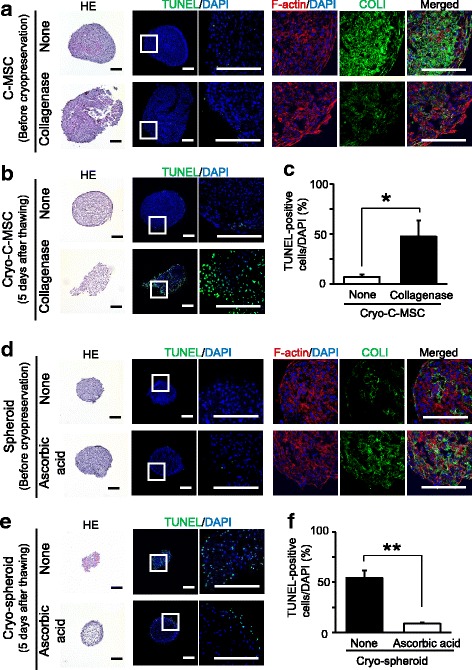
Protective role of type I collagen (COLI) against cryopreservation-induced damage in MSC spheroids and C-MSCs. **a,b** Clumps of mesenchymal stem cell/extracellular matrix complexes (C-MSCs) were treated with or without collagenase for 15 min before cryopreservation. **d,e** MSC spheroids were generated with growth medium in the presence or absence of 50 μg/mL l-ascorbic acid. Histological analysis of cell constructs before cryopreservation (**a,d**) or at 5 days after thawing (**b,e**). Serial sections were stained with hematoxylin and eosin (HE) and TUNEL as indicated. Scale bars = 200 μm. Magnified images of the boxed regions are shown in the panels to the immediate right. In addition, the samples before cryopreservation were immunostained with an anti-type I collagen antibody (green). Nuclei and F-action were counterstained with 4′,6-diamidino-2-phenylindole (DAPI; blue) and Alexa594-conjugated phalloidin (red), respectively. Scale bars = 200 μm. **c,f** The percentage of TUNEL-positive apoptotic cells in C-MSCs (**c**) or MSC spheroids (**f**) 5 days after thawing. Values represent means ± SD of four cultured cell constructs. Similar results were obtained from three independent experiments. *p < 0.05, **p < 0.01, by *t* test

Cell spheroids, which were not stable against the damage from cryopreservation, are mainly composed of cell–cell contacts, not ECM [22]. On the other hand, C-MSCs consisted of the cells and abundant ECM, such as type I collagen [8]. This discrepancy led us to make a hypothesis that abundant type I collagen plays a protective role against damage by cryopreservation in C-MSCs. To test this tentative hypothesis, we tested the effect of collagenase treatment on C-MSC cryopreservation. Collagenase treatment for 15 min did not induce TUNEL-positive apoptotic cells, though type I collagen expression levels were dramatically reduced in C-MSCs (Fig. 4a). Importantly, as we expected, C-MSCs treated with collagenase suffered collapse of their round shape after cryopreservation and the numbers of TUNEL-positive apoptotic cells increased (Fig. 4b, c). These findings clearly suggest that type I collagen in C-MSCs plays a protective role against damage from cryopreservation. To support this finding, we examined the effect of type I collagen in cell spheroids on cryopreservation using ascorbic acid which is known to facilitate type I collagen production in MSCs [23]. The cellular spheroids generated by growth medium containing ascorbic acid showed a round shape similar to that of normal spheroids, although type I collagen expression levels were obviously higher (Fig. 4d). The spheroids generated with ascorbic acid treatment retained their spheroidal shape after cryopreservation and few apoptotic cells were detectable only at the edge of the cellular construct (Fig. 4e, f). Taken together, plenty of ECM such as type I collagen may be protective of the 3D structure of cellular constructs against damage from cryopreservation.

### Transplantation of cryopreserved C-MSCs without any artificial scaffold can induce rat calvarial bone regeneration

**Fig. 5:**
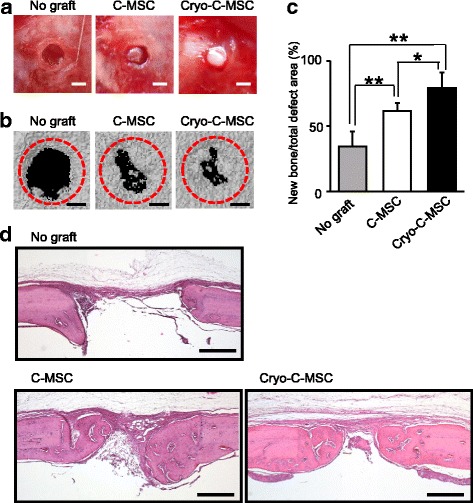
Both C-MSC and cryopreserved C-MSC transplantation induces rat calvarial bone regeneration. **a** Cultured clumps of mesenchymal stem cell/extracellular matrix complexes (C-MSCs) or cryopreserved C-MSCs (cryo-C-MSC) were transplanted into a rat calvarial defect 1.6 mm in diameter with no artificial scaffold. Scale bars = 1.0 mm. **b** Micro-CT images of bone regeneration by C-MSCs or cryo-C-MSCs. The calvarial defect area was scanned by micro-CT on day 28. Scale bars = 500 μm. **c** The volume of mineralized tissue on day 28 was quantified. Data are the mean ± SD of four rats per group. *p < 0.05, **p < 0.01, by ANOVA. **d** All rats were sacrificed 28 days after implantation of C-MSCs and the calvarial bones were fixed. Coronal sections were obtained and stained with H&E. Scale bars = 500 μm. The photographs are representative of four independent experiments

**Fig. 6:**
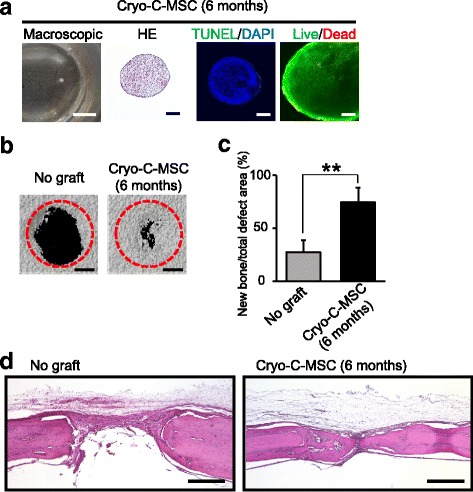
Prolonged cryopreservation does not reduce cell viability and bone regenerative properties of C-MSCs. Clumps of mesenchymal stem cell/extracellular matrix complexes (C-MSCs) were cryopreserved for 6 months as described in the Methods. **a** Left panel shows macroscopic images of cryopreserved C-MSCs (cryo-C-MSC) in a 24-well culture plate. Scale bar = 4 mm. Serial sections were stained with hematoxylin and eosin (HE) and TUNEL as indicated. In addition, to monitor the cell viability, live (green) and dead cells (red) were distinguished by using a LIVE/DEAD Viability/Cytotoxicity kit. Scale bars = 200 μm. C-MSCs cryopreserved for 6 months were transplanted into a rat calvarial defect 1.6 mm in diameter with no artificial scaffold. **b** Micro-CT images of bone regeneration. The calvarial defect area was scanned by micro-CT on day 28. Scale bars = 500 μm. **c** The volume of mineralized tissue on day 28 was quantified. Data are the mean ± SD of four rats per group. *p < 0.05, by ANOVA. **d** All rats were sacrificed 28 days after the implantation of cryo-C-MSCs and the calvarial bones were fixed. Coronal sections were obtained and stained with H&E. Scale bars = 500 μm. The photographs are representative of four independent experiments

Previously, we reported that C-MSC implantation with no artificial scaffold can induce rat calvarial bone regeneration [8]. Accordingly, we examined whether cryopreserved C-MSCs also possess bone regenerative capacity. C-MSCs or cryopreserved C-MSCs were directly grafted into rat calvarial defects without any artificial scaffold (Fig. 5a). Compared with the no graft group, both C-MSC and cryopreserved C-MSC transplantation induced similar levels of mineralized tissue formation (Fig. 5b, c). Moreover, consistent with these micro-CT findings, both C-MSC and cryopreserved C-MSC implantation facilitated bone regeneration in the lesion area (Fig. 5d). These findings implied that cryopreserved C-MSCs possess bone regenerative capacity identical to normal C-MSCs. Finally, we investigated the effect of prolonged cryopreservation on cell viability and bone regenerative property in C-MSCs. C-MSCs cryopreserved for 6 months retained a round shape and did not increase the number of dead cells (Fig. 6a). Thawed C-MSCs that had been frozen for 6 months and cultured for 5 days were then grafted into a rat calvarial defect model. As a result, cryopreserved C-MSC transplantation induced successful bone regeneration as evidenced by micro-CT and H&E staining (Fig. 6b–d). These data implied that prolonged cryopreservation did not reduce the bone regenerative capacity of C-MSCs.

## Discussion

In this present study, we demonstrated that cryopreservation did not reduce cell viability or osteogenic differentiation potential in C-MSCs in vitro. Moreover, cryopreserved C-MSC transplantation induced bone regeneration in a rat calvarial defect model. These findings imply a great advantage for C-MSCs in their clinical application since successful cryopreservation of C-MSCs can omit the preparation process and enable a reliable supply of good quality-controlled material as needed, which increases the reliability of the therapeutic efficacy of C-MSC transplantation. In addition, cryopreservation of C-MSC will guarantee more beneficial effects on allogenic transplantation therapy. Cryopreservation of allo-C-MSCs should eliminate the culture process associated with the isolation and expansion of auto-MSCs from patients, which could result in savings in terms of the cost and culture period. Cryopreservation of allo-C-MSCs will also facilitate logistical transport of the cellular products to treatment centers and allow sufficient time for screening for contaminations or transmissible diseases. Importantly, we have reported previously that C-MSCs treated with IFN-γ may be applicable for clinical allogenic bone regenerative therapy due to their highly regulated immunomodulatory properties [10]. Taken together, the combination of cryopreservation and IFN-γ treatment may lead to reliable “off-the-shelf” C-MSC therapy for bone regeneration.

For some time now it has been accepted that the cooling rate [24] and the composition of cryoprotectants [25] are involved in damage caused by cryopreservation. Importantly, MSCs show relatively better resilience during cryopreservation than various kinds of cells that require optimal cooling rates and cryoprotectants. Therefore, slow freezing in the presence of a cryoprotectant such as 10% DMSO is the traditional approach for the cryopreservation of MSCs as a single suspension. In addition, several improved cryopreservation protocols using controlled freezing systems and cytoprotective reagents have been reported for single-cell suspensions [26]. Despite these advanced MSC cryopreservation studies, it is still a big challenge to cryopreserve 3D MSC scaffold constructs with maintained cellular viability and function, which would be an attractive approach for regenerative cell therapy to allow immediate supply of the cell constructs with ready-to-use characteristics. The difficulty is assumed to be due to the fact that cell–substrate and cell–cell contacts are more sensitive to cryoinjury, followed by cell detachment and death [27–29], which is not found in the cryopreservation of single-cell suspensions. Only a few studies have investigated the cryopreservation of adherent MSCs on synthetic scaffolds [30, 31], although the tissue regenerative properties of cryopreserved MSC constructs were not determined. ECM produced by MSCs themselves, which were composed of C-MSCs, might be better scaffolds for cell adhesion compared with artificial ones, and thereby result in successful cryopreservation. In fact, Zeitouni et al. demonstrated that scaffolds mainly consisting of MSC-derived ECM proteins exhibited good cell-binding properties [32]. Importantly, their subsequent study showed that the composition of osteogenically enhanced MSCs and the self-produced scaffold could be cryopreserved for 7 days without a reduction in cell viability or bone regenerative properties [33]. More specifically, ECM produced by MSCs might be a useful material for the cryopreservation of 3D MSC scaffold constructs, which could shed light on novel approaches in regenerative medicine.

In this regard, type I collagen could be a critical component in such cryoprotective ECM produced by the cells themselves since we demonstrated that cryopreservation of C-MSCs pretreated with collagenase disrupted their 3D structural integrity with the induction of cell death (Fig. 4). In addition, cell spheroids generated with ascorbic acid treatment, which stimulated type I collagen production, retained a round 3D structure and showed few apoptotic cells after cryopreservation (Fig. 4), suggesting the cryoprotective role of type I collagen produced by the cells. Of note, although the cell sheet tested in this present study could not retain its structural integrity and cell viability after cryopreservation, Li et al. demonstrated that a sheet of periodontal ligament stem cells (PDLSCs) was resistant to cryopreservation [34]. However, we deemed that this discrepancy does not oppose but supports our finding because the PDLSC sheet, which was generated by treatment with sufficient ascorbic acid and a long culture period (10–14 days) using a 60-mm culture plate [34], was obviously bigger (around 15 mm in diameter) and could contain higher amounts of ECM proteins than the cell sheet tested in our present study. Taken together, the abundant self-produced ECM, including type I collagen, may play a key role in the successful cryopreservation of 3D cell constructs.

The 3D structure of cryopreserved MSC spheroids seemed to be damaged after 1 day of thawing, although the round shape was retained and the cells still survived (Fig. 3c). However, it was noted that TUNEL-positive apoptotic cells appeared in the cell construct accompanied by the disruption of its 3D structural integrity after 5 days thawing (Fig. 3d). These findings indicated that freezing damaged not the cell viability but the physicality of the ECM, which formed the 3D cell construct. Of note, it is a fact that the loss of cell attachment to the ECM or cell–cell contacts induces a specific type of apoptosis, termed anoikis [35]. Briefly, the apoptosis observed in cryopreserved MSC spheroids (Fig. 3d) or cryopreserved C-MSCs that were pretreated with collagenase (Fig. 4b) may be attributed to anoikis. Therefore, the success of cryopreservation of MSC 3D constructs including cell sheets, spheroids, and C-MSCs may depend on the stability of the ECM, which circumvents the problems of anoikis. Further basic studies focusing on not only cells but also protein freezing may open new avenues for novel approaches to regenerative medicine using cryopreserved MSCs.

The osteogenic differentiation potential of MSCs is one of the main advantages for bone regenerative medicine. Although cryopreservation did not affect MSC-specific marker expression patterns or cell viability in C-MSCs, osteogenic differentiation capacity was higher in cryopreserved C-MSCs than in normal C-MSCs (Fig. 2b–d). This tendency was also observed in the in vivo study (Fig. 5c). It should be noted that cryopreserved C-MSCs were apparently larger compared with C-MSCs without cryopreservation after a 10-day culture period (Fig. 2d). In general, C-MSCs can contract and become smaller in a time-dependent manner during culture due to ECM elasticity [8]. However, cryopreserved C-MSCs did not shrink but increased the amount of void spaces in ECM, and thereby grew in size. These findings suggested that cryopreservation affects the ECM structure in C-MSCs. To support this finding, a previous study demonstrated that cryopreservation impacts the structure of the ECM and increases the fragmented appearance of the histoarchitecture in heart valve leaflets [36]. In addition, cryopreservation elevated OIM-induced calcium deposition, as indicated by Alizarin Red staining. These findings may imply the possibility that the freezing process influences the physiological characteristics of the ECM which might be associated with osteogenic differentiation potential. Indeed, it is known that the ECM provides structural and functional proteins that regulate cellular behavior and lineage-specific differentiation to facilitate osteogenesis [37, 38]. Accordingly, although the precise molecular mechanisms remain elusive, the higher osteogenic property in cryopreserved C-MSCs might be attributed to the change of ECM structure caused by cryopreservation. Additional investigations are required.

In this present study, C-MSC or cryopreserved C-MSC transplantation induced bone regeneration in 1.6-mm diameter calvarial defects. However, it might be hard for both of them to exert successful bone regeneration in a critical-sized bone defect model because we have previously reported that transplantation of C-MSCs cultured with growth medium for 5 days failed to induce bone regeneration in a 3.0-mm diameter calvarial defect [8]. On the other hand, OIM-treated C-MSC implantation induced acceptable bone regeneration in the created 3.0-mm calvarial defect [8]. Therefore, because cryopreserved C-MSCs cultured with OIM showed higher osteogenic capacity (Fig. 2b–d), the combination of cryopreservation and OIM might be an appropriate way to apply C-MSCs for clinical bone regenerative cell therapy.

Nevertheless, load-bearing bone defect cases in clinical orthopedics still seem to be a challenge for C-MSC transplantation therapy because C-MSCs may be too weak to retain the load pressure at the defect site. Accordingly, to treat the load-bearing bone defect by C-MSC transplantation the combined use of some artificial scaffold may be needed.

## Conclusion

In conclusion, C-MSCs which contain abundant type I collagen retained cell viability and bone regeneration properties through the cryopreservation process. Therefore, cryopreserved C-MSCs, which can circumvent the preparation process before transplantation and provide standardized material on demand, may represent a novel bone regenerative cell therapy. In addition, if allogenic MSCs can be utilized, cryopreserved C-MSCs may lead to reliable “off-the-shelf” MSC transplantation therapy.

## Abbreviations

3D: Three-dimensional
ALP: Alkaline phosphatase
CD: Cluster of differentiation
C-MSC: Clumps of mesenchymal stem cell/extracellular matrix complex
CT: Computed tomography
DAPI: 4′,6-Diamidino-2-phenylindole
DMEM: Dulbecco’s modified Eagle’s medium
DMSO: Dimethyl sulfoxide
ECM: Extracellular matrix
EthD-1: Ethidium homodimer
FBS: Fetal bovine serum
FITC: Fluorescein isothiocyanate
H&E: Hematoxylin and eosin
IFN: Interferon
MSC: Mesenchymal stem cell
OIM: Osteoinductive medium
PBS: Phosphate-buffered saline
PDLSC: Periodontal ligament stem cell
TUNEL: TdT-mediated dUTP nick end labeling
